# Adsorption of Cr(VI) and Speciation of Cr(VI) and Cr(III) in Aqueous Solutions Using Chemically Modified Chitosan 

**DOI:** 10.3390/ijerph9051757

**Published:** 2012-05-07

**Authors:** Jun Dai, FengLian Ren, ChunYuan Tao

**Affiliations:** 1 College of Chemistry and Chemical Engineering, Central South University, Changsha 410083, Hunan, China; Email: deardaijun@sina.com; 2 College of Chemistry and Chemical Engineering, JiuJiang University, Jiujiang 332005, Jiangxi, China; Email: taochunyuan@sina.com

**Keywords:** grafting chitosan, speciation, flame atomic absorption spectrometry, chromium

## Abstract

A new type of grafting chitosan (CTS) was synthesized using 2-hydroxyethyl- trimethyl ammonium chloride (HGCTS). The adsorption of Cr(VI) on HGCTS was studied. The effect factors on adsorption and the adsorption mechanism were considered. The results indicated that the HGCTS could concentrate and separate Cr(VI) at pH 4.0; the adsorption equilibrium time was 80 min; the maximum adsorption capacity was 205 mg/g. The adsorption isotherm and kinetics were investigated, equilibrium data agreed very well with the Langmuir model and the pseudo second-order model could describe the adsorption process better than the pseudo first-order model. A novel method for speciation of Cr(VI) and Cr(III) in environmental water samples has been developed using HGCTS as adsorbent and FAAS as determination means. The detection limit of this method was 20 ng/L, the relatively standard deviation was 1.2% and the recovery was 99%~105%.

## 1. Introduction

Chromium is one of the major trace heavy metal pollutants in the environment. Chromium in environmental waters typically comes from industrial pollution sources, including tanning factories, steel works, wood preservation and artificial fertilizers. It is widely known that the toxicity and biological activity of the element not only depends on the total amount, but also on its chemical form [1,2]. Chromium species exist mainly in two different oxidation states in environmental water: Cr(VI) and Cr(III), which have contrasting physiological effects. Cr(III) is considered as an essential trace element for the maintenance of an effective glucose, lipid, and protein metabolism in mammals [3]. On the other hand, Cr(VI) can be toxic for biological systems and cancerogenic in humans [4,5]. Therefore, speciation of Cr(VI) and Cr(III) is necessary for evaluating the toxicological behavior of chromium. In recent years, more and more papers [6,7,8] about chromium speciation were reported.

Chromium is usually present at trace levels in environmental water samples, therefore very sensitive techniques are used for the determination of chromium in water samples such as flame atomic absorption spectrometry (FAAS) [9], graphite furnace atomic absorption spectrometry (GFAAS) [10], inductively coupled plasma-atomic emission spectrometry (ICP-AES) [11] and inductively coupled plasma-mass spectrometry (ICP-MS) [12]. But these modern instrumental techniques can only yield total amount of chromium. As a result, a preliminary species preconcentration and separation step is often required. Selection of an high efficient preconcentration reagent shows its importance here. 

The methods used for preconcentration and separation include chemical precipitation [13], ion exchange [14], solvent extraction [15] and adsorption [16]. Among these methods, adsorption has been proved to be an efficient and economical technique. Activated carbon and silica gel are the two most popular adsorbents [17] in trace element analysis, but they are relatively expensive materials since the higher the quality, the greater they cost. The search for alternative adsorbents has intensified in recent years. At present, the focus is on chitosan. Chitosan is prepared from chitin by deacetylating its acetamido groups to different degrees. Chitosan has both hydroxyl and amine groups that can be chemically modified [18,19,20] by reactions such as cross-linking, grafting, alkylating, esterification, *etc*. Chemical modifications can offer a wide spectrum of tools to enhance the sorption properties of chitosan for metals. They may increase the chemical stability of the sorbent in acid media and, especially, decrease the solubility in most mineral and organic acids. They also increase its resistance to biochemical and microbiological degradation. The use of chitosan and its derivatives as adsorbents for metal ions [21], as flocculants [22], and carriers for medicine [23] have been reported, but reports about using chitosan and its derivatives to preconcentrate and separate Cr(VI) and Cr(III) in environmental water samples are rare.

In this work, a new type of chitosan grafted with 2-hydroxyethyltrimethyl ammonium chloride (HGCTS) was synthesized, which has quaternary ammonium groups. Then, using HGCTS as sorbent, and FAAS as determination means, the adsorption of Cr(VI) on HGCTS was studied, and a novel method for speciation of Cr(VI) and Cr(III) in environmental water samples was developed.

## 2. Experimental

### 2.1. General

Chitosan (deacetylation degree 90%) and 2-hydroxyethyltrimethyl ammonium chloride were purchased from Shanghai National Reagent Company (Shanghai, China). Acetic acid (analytical grade, Beijing Chemical Engineering Factory, Beijing, China), alcohol (analytical grade, Third Reagent Factory of Shanghai, Shanghai, China) and isopropanol (analytical grade, Shanghai Yonghua Chemistry Reagent Factory, Shanghai, China) were provided by the chemistry laboratory of JiuJiang University (Jiujiang, China). 0.1 mol/L HCl and 0.1 mol/L NaOH were used to control the pH values of the solutions, 1 g/L Cr(VI) and Cr(III) stock solution were prepared by dissolving the appropriate amount of K_2_Cr_2_O_7_ and CrCl_3_·6 H_2_O in doubly distilled water, which was used throughout the entire experiment. Chromium was determined on a FAAS model AA6300C (Shimadzu, Kyoto, Japan) with a chromium hollow cathode lamp and deuterium background correction. Its operating conditions are given in Table 1. pH values was measured on a model PHS-3C pH meter (Shanghai Precision Instrument Company, Shanghai, China). The IR spectrum of the product was recorded on a model Vertex70 infrared spectrometer (Bruker, Karlsruhe, Germany) using KBr discs. The Scanning Electron Microscope (SEM) imaging was performed on a SEM model VegaII (Tescan, Brno, Czech). The surface areas of the CTS and HGCTS were measured on a model ASAP 2010 surface analyzer (Micromeritics, Atlanta, USA) with the Brunauer-Emmett-Teller (BET) method.

**Table 1:** FAAS operating conditions.

FAAS parameters	
Lamp current(mA)	10
Slit width(nm)	0.7
Flow rate of acetylene(L/min)	2.8
Flow rate of air(L/min)	15.0
Analytical wavelength(Cr, nm)	357.9

### 2.2. Preparation of 2-Hydroxyethyltrimethyl Ammonium Chloride Grafted Chitosan

**Scheme 1:**
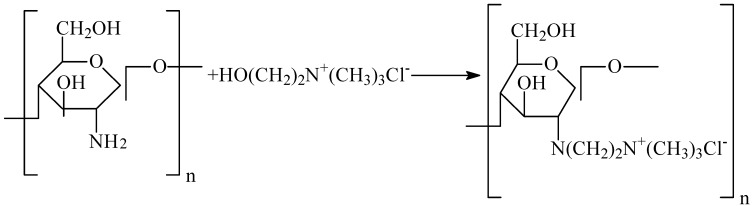
Preparation of HGCTS.

The preparation of the HGCTS grafted chitosan is outlined in Scheme 1.

CTS (3.0 g) was dissolved in 1% aqueous acetic acid (60 mL) for 30 min. Alcohol (10 mL) was added to dilute the solution. Then 2-hydroxyethyltrimethyl ammonium chloride (9.0 g) and isopropanol (50.0 mL) were added to the solution in turn. The mixture was stirred with water bath heating at 70 °C for 8 h until the product was obtained. The solid product obtained was filtered off and washed several times with ethanol, followed by distilled water. Then the product was dried at 60 °C in a vacuum drying oven.

The degree of substitution of free amino groups was represented by the concentration of quaternary amine groups on the HGCTS, and the concentration of quaternary amine groups was estimated by colloid titration [24]. HGCTS (0.1 g) was dissolved in distilled water (100 mL), and an aliquot of this solution (10 mL) was transferred into a conical flask, toluidine blue solution (0.1%, 0.5 Ml0 was added as indicator, AND then the sample solution was titrated with polyvinyl alcohol potassium sulfate standard solution. The end point was obtained when the color of the solution changed from blue to red. The volume of the titrant was recorded. At the same time, the blank solution titration was performed. The concentration of quaternary amine groups was calculated by the equation below: 


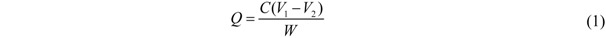


where *Q* (mmol/g) is the concentration of quaternary amine groups, *C* (mol/L) is the concentration of titrant, *V*_1_ and *V*_2_ (mL) are the volume of the solution of titrant for the sample and blank solution titration respectively, *W* (g) is the weight of HGCTS. The concentration of quaternary amine groups was found to be 3.65 mmol/g.

### 2.3. Effect of PH

The effect of pH on adsorption of Cr(VI) and Cr(III) was studied in pH range 1.0–10.0 at 25 °C by shaking dry HGCTS (10 mg) with Cr(VI) and Cr(III) solution (200 mL, 6 μg/mL) for 80 min at 300 rpm. The desired pH was adjusted using 0.1 mol/L HCl and 0.1 mol/L NaOH. After filtration, the concentration of Cr(VI) and Cr(III) in solution was determined by FAAS.

### 2.4. Kinetics of Adsorption

Kinetic studies were conducted by placing HGCTS (10 mg) in a 250 mL flask containing Cr(VI) solution (200 mL, 6 μg/mL) at pH 4.0 and 25 °C. The mixture was stirred by a magnetic stirrer at 300 rpm. Samples of solution (10 mL) were withdrawn at scheduled time intervals and analyzed for Cr(VI) concentration after filtration.

### 2.5. Adsorption Isotherm

At 25 °C, 35 °C and 45 °C, a series of different concentrations of Cr(VI) standard solutions (200 mL) were prepared. The pH of the solution was adjusted to 4.0. HGCTS (10 mg) was added to the solution which was stirred at 300 rpm for 80 min, then filtered. After filtration, the concentration of Cr(VI) was determined by FAAS. The adsorption capacity was calculated according to:


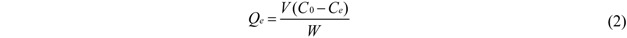


where *Q*_e_ (mg/g) is the adsorption capacity, *C*_0_ (μg/mL) is the initial concentration of Cr(VI), *C*_e_ (μg/mL) is the equilibrium concentration of Cr(VI), *V* (mL) is the volume of the solution of Cr(VI), *W* (mg) is the weight of HGCTS added.

### 2.6. Adsorption, Desorption and Determination of Cr(VI)

HGCTS (10 mg) was added to Cr(VI) solution (200 mL) at 25 °C. Then the pH of the solution was adjusted to be 4.0 with 0.1 mol/L HCl and 0.1 mol/L NaOH. The solution was filtered off after surging for 80 min. The HGCTS was washed several times with doubly distilled water. NaOH (0.1 mol/L, 5 mL) was used to elute Cr(VI) from the HGCTS. The final volume of eluting solution was 10 mL. The concentration of Cr(VI) was determined by FAAS

### 2.7. Determination of Total Cr and Cr(III)

1% K_2_S_2_O_8_ aqueous solution (5 mL) was added to a water sample to oxidize Cr(III) to Cr(VI) with heating. Then the procedure to determine total Cr was applied as described above. Cr(III) concentration was obtained as the respective difference between total Cr and Cr(VI) .

## 3. Results and Discussion

### 3.1. Characterization of HGCTS

#### 3.1.1. Physical Characteristics

Some physical properties of CTS and HGCTS were measured in this work, and the results are listed in Table 2. It is useful for us to know the studied materials.

**Table 2:** Physical properties of CTS and HGCTS.

Materials	Surface area (m^2^/g)	Average pore diameter (nm)
CTS	1.45	5.10
HGCTS	1.75	6.82

#### 3.1.2. FTIR Spectrum

**Figure 1:**
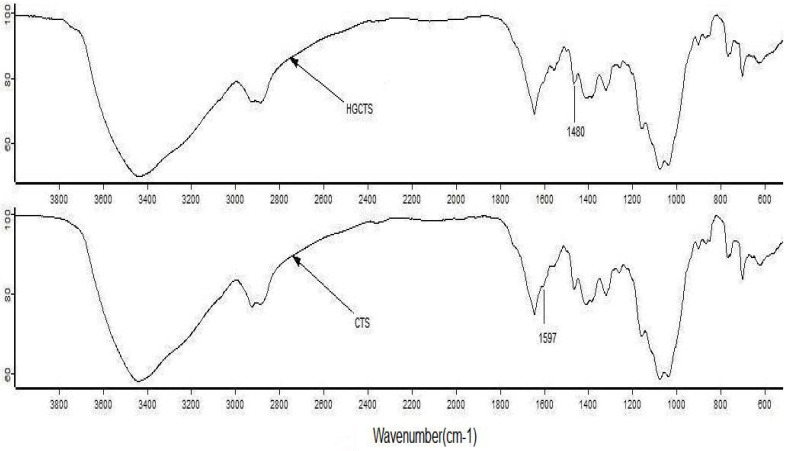
IR spectra of CTS and HGCTS.

The IR spectrum of HGCTS shown in Figure 1 resembled closely that of CTS. The most striking difference between the two spectra is that the band at 1,597 cm^−1^ [24] attributed to a primary amine (N–H) of CTS disappeared and the band at 1,480 cm^−1^, which corresponds to the methyl groups of the quaternary hydrogen [25] appeared in the HGCTS spectrum. This indicated that the amine of CTS had reacted with 2-hydroxyethyltrimethyl ammonium chloride to form HGCTS.

#### 3.1.3. SEM Image

**Figure 2:**
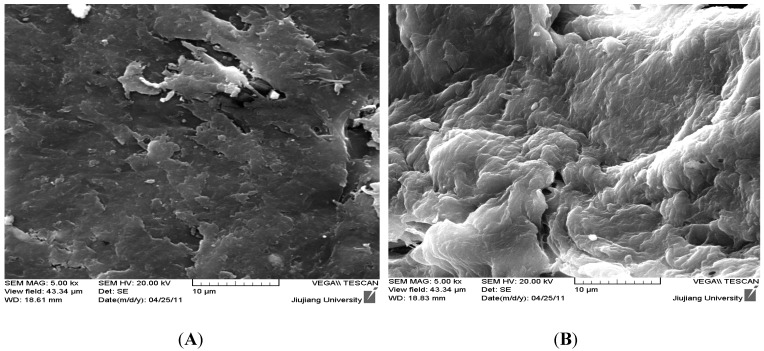
SEM images of (**A**) CTS and (**B**) HGCTS.

Figure 2 shows the SEM images of CTS and HGCTS. The images show that the surface of CTS was relatively smooth and the structure of CTS was compact. The surface of HGCTS was rough, with a lot of cavities on the surface and the structure of HGCTS was uncompact. The reticular structure of HGCTS could enlarge its surface area (the surface area of HGCTS was 1.75 m^2^/g, whereas that of CTS was 1.45 m^2^/g) and enhance its adsorption ability.

### 3.2. Effect of pH on Adsorption of Cr(VI) and Cr(III)

**Figure 3:**
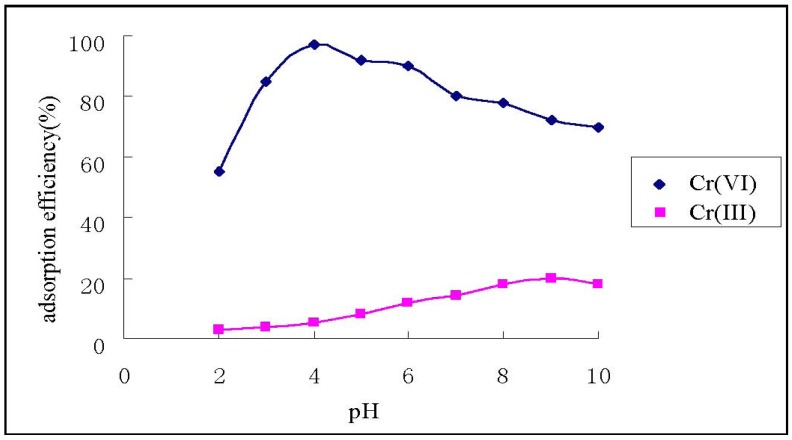
Effect of pH on the adsorption of Cr(VI) and Cr(III).

Figure 3 shows that HGCTS adsorbed Cr(VI) strongly under acid conditions. The adsorption efficiency of Cr(VI) achived its maximum value (97%) at pH 4.0, whereas the adsorption efficiency of Cr(III) was 5%. Thus, at pH 4.0, the separation of Cr(VI) and Cr(III) could be realized. 

The adsorption mechanism of Cr(VI) on chitosan and its derivatives is electrostatic attraction and ion exchange, whereas which of Cr(III) is chelation [26]. The Cr(III) cation has an empty orbit, and the amine group on HGCTS has an isolated pair of electrons, so when HGCTS reacts with Cr(III), a chelated complex was formed by coordination. In acid solutions, Cr(VI) exists mainly as HCrO_4_^–^ and Cr_2_O_7_^2–^, which have negative charges. Amido groups on HGCTS react with H^+^, producing –NH_3_^+^, groups which adsorb Cr(VI) anion strongly through electrostatic attraction. When Cr(VI) anion reaches the surface of HGCTS, an ion exchange reaction occurred, that could be represented as follows:


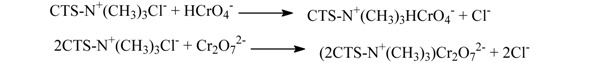


On the other hand, more than 90% of the active sites of CTS are protonated in the low pH range [26], the chelation of Cr(III) becomes weak, and the adsorption of Cr(III) is less probable. The observed decrease in the uptake value of Cr(VI) at pH < 4.0 may be attributed to the higher concentration of Cl^-^ which competes with the chromate anions’ interaction with the protonated amine active sites [25]. Above pH = 4.0, the adsorption efficiency of Cr(VI) decreases as pH increases. This may be explained on the basis of the lower extent of protonated amido groups with rising pH.

### 3.3. Kinetic Studies

**Figure 4:**
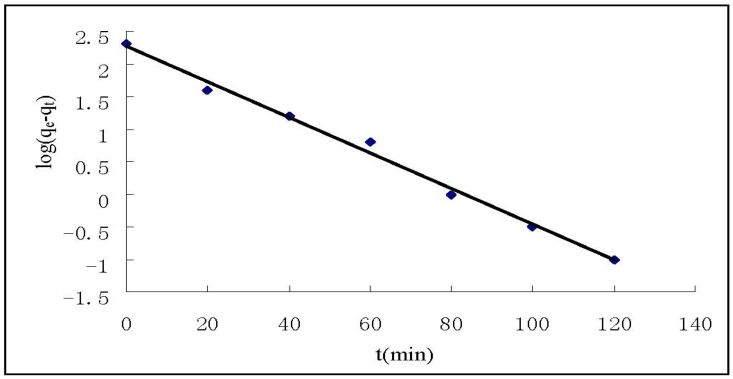
Pseudo first-order kinetic plots for the adsorption of Cr(VI).

**Figure 5:**
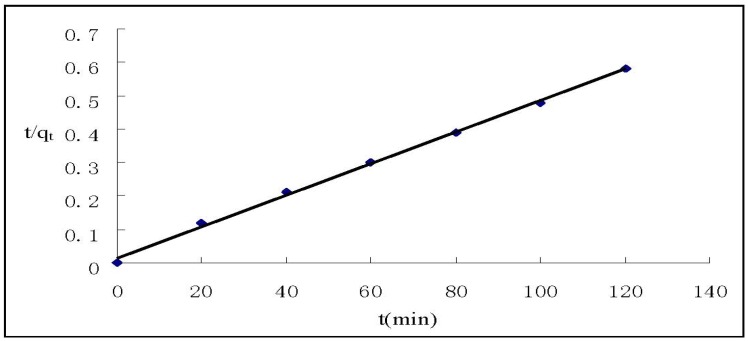
Pseudo second-order kinetic plots for the adsorption of Cr(VI).

The kinetic study results show that the adsorption of Cr(VI) on HGCTS increased with increasing contact time and attained equilibrium at about 80 min. In order to investigate the adsorption kinetic process, the pseudo first-order and pseudo second-order kinetic models were applied in this study. The pseudo first-order model is expressed as [27]:


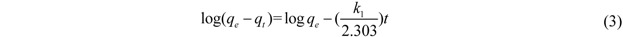


where q_e_ and q_t_ (mg/g) are the amounts of Cr(VI) adsorbed on HGCTS at equilibrium and at time t, respectively, and k_1_ is the pseudo first-order rate constant (min^−1^) of adsorption. The rate constant, k_1_ and correlation coefficient, R^2^ were determined by plotting log(q_e_−q_t_) *versus* t. The pseudo second-order model is expressed as [28]:


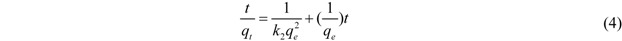


where k_2_ is the pseudo second-order rate constant(g mg^−1^ min^−1^) of adsorption. The rate constant, k_2_ and correlation coefficient, R^2^ were determined by plotting t/q_t_ *versus* t. The kinetic models for Cr(VI) adsorption are shown in Figure 4 and Figure 5. The parameter values of the kinetic models are presented in Table 3. According to Figure 4 and Figure 5 and based on the correlation coefficients in Table 3, the pseudo second-order model could better describe the adsorption of Cr(VI) on HGCTS than the pseudo first-order model. This suggests that the rate-limiting step may be chemical adsorption [16]. 

**Table 3:** Kinetic parameters for Cr(VI) adsorption on HGCTS.

Metal ion	Pseudo first-order		Pseudo second-order	
	k_1_ (min^−1^)	R^2^	k_2_ (g mg^−1^ min^−1^)	R^2^
Cr(VI)	0.063	0.9935	0.0016	0.9983

### 3.4. Adsorption Isotherm of Cr(VI) on HGCTS

**Figure 6:**
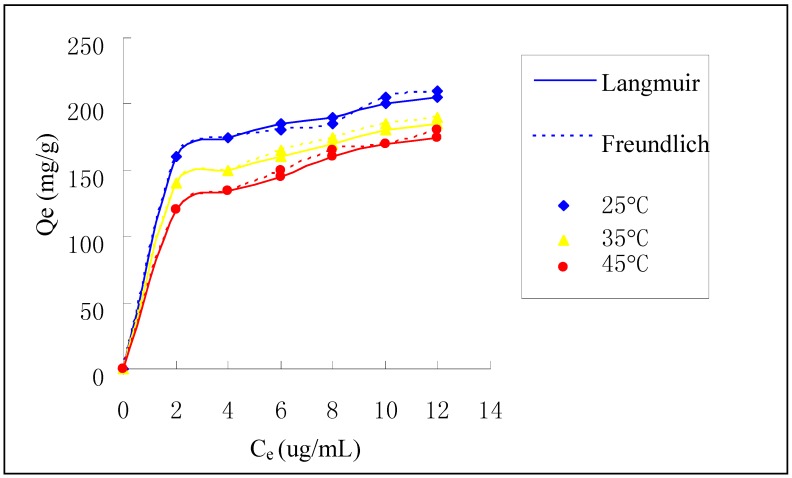
Adsorption isotherm of Cr(VI) on HGCTS.

Figure 6 shows the adsorption isotherm of Cr(VI) on HGCTS and the maximum adsorption capacity is about 205 mg/g. The experimental data in Figure 6 were treated by the Langmuir and Freundlich equations to examine the relation between sorption and metal ion concentration at equilibrium. The Langmuir model, which is widely used for monolayer sorption on a surface, is presented as:


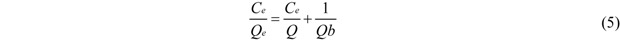


where Q_e_ (mg/g) is the adsorption capacity of Cr(VI) at equilibrium concentration, Q (mg/g) is the maximum adsorption capacity, C_e_ (μg/mL) is the equilibrium concentration of Cr(VI), b (mL/μg) is the Langmuir constant. Q and b can be calculated by plotting C_e_/Q_e_ *versus* C_e_. For the Langmuir model, it is estimated by a dimensionless separation factor whether the sorption is favorable or not. The separation factor, R_L_ is defined as:


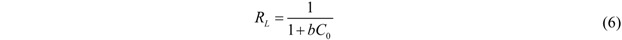


where C_0_ (μg/mL) is the initial concentration of Cr(VI), b (mL/μg) is the Langmuir constant. Values of 0 < R_L_ < 1 indicates that the sorption is favorable. The values of R_L_ in this study lie in the range of 0.017 and 0.148 for Cr(VI), which shows that the adsorption of Cr(VI) on HGCTS is favorable. 

The Freundlich model, which is widely used for sorption on a heterogeneous surface, is given by:


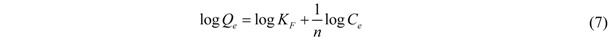


where K_F_ and n are Freundlich constants related to adsorption capacity and intensity, respectively. K_F_ and n can be determined from a linear plot of logQ_e_ *versus* logC_e_. The constants of two model along with correlation coefficient (R^2^) values are presented in Table 4. It is found that the Langmuir model fit the data better than the Freundlich model, which indicates that the adsorption of Cr(VI) on HGCTS is a type of monolayer sorption.

**Table 4:** Parameters of Langmuir and Freundlich models for Cr(VI) adsorption.

Temperature(°C )	Langmuir model			Freundlich model		
	Q (mg/g)	b (mL/ug)	R^2^	K_F_ (mg/g)	n	R^2^
25	204	2.88	0.9978	147.2	7.34	0.9672
35	189	1.61	0.9912	122.5	5.75	0.9720
45	181	1.19	0.9852	100.5	4.29	0.9848

To obtain the thermodynamic parameters of the adsorption, the values of b at different temperatures were processed according to van’t Hoff equation:


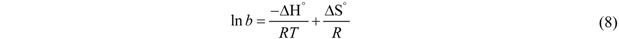


where ∆H° and ∆S° are enthalpy and entropy changes, respectively, R is the universal gas constant (8.314 J/mol·K) and T is the absolute temperature (in Kelvin). Plotting lnb against 1/T gives a straight line with slope and intercept equal to *–*∆H°/R and ∆S°/R, respectively. Gibbs free energy of adsorption (∆G°) was calculated by the following equation:


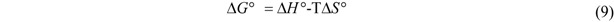


The values of ∆H°, ∆S° and ∆G° are given in Table 5. The negative values of ∆H° indicate the adsorption process is exothermic and the negative values of ∆G° show that it is spontaneous. 

**Table 5:** Thermodynamic parameters for Cr(VI) adsorption.

Temperature (°C)	∆H° (kJ/mol)	∆G° (kJ/mol)	T∆S° (kJ/mol)
25	−24.58	−22.71	−1.87
35	−24.58	−21.96	−2.62
45	−24.58	−21.21	−3.37

### 3.5. Effects of Foreign Ions

The influences of some ordinary ions typically present in water were investigated. Various amounts of ions were added to a 2 μg/mL Cr(VI) standard solution (50 mL) and the described procedure was followed. 

The results of this study are given in Table 6, from which we know that the major matrix ions show no obvious interference with the adsorption and determination of Cr(VI).

**Table 6:** Influences of some foreign ions on the recoveries of Cr(VI) (*n* = 3).

Ion	Added as	Concentration (μg/mL)	Recovery (%)
Na^+^	NaCl	1,000	99.6
K^+^	KCl	1,000	99.5
Mg^2+^	MgCl_2_	1,000	98.8
Ca^2+^	CaCl_2_	300	98.5
Zn^2+^	ZnCl_2_	100	98.2
Cu^2+^	CuCl_2_	50	97.4
Fe^3+^	FeCl_3_	50	97.8
Cl^−^	NaCl	1,000	96.8
NO_3_^−^	KNO_3_	1,000	96.3

Data are expressed as mean of three replicates.

### 3.6. Characteristics and Application of the Proposed Method

Under the optimal experimental conditions, the ten replicates of the blank solution were determined. The detection limit, based on three times the standard deviation of the blank, was 20 ng/L and the relative standard deviation was 1.2% (n = 6). Table 7 compares the adsorption capacity of HGCTS used in this method with other adsorbents reported in the literature. From these sources, we can see that the adsorption capacity of HGCTS is comparable to those adsorbents reported in the literatures.

**Table 7:** Comparison of adsorption capacity for Cr(VI) on HGCTS with other adsorbents.

Adsorbents	Adsorption Capacity (mg/g)	Refs
GCCTS	215	[26]
Fe-CCTS	295	[29]
MCCTS	150	[25]
HGCTS	205	This work

GCCTS: Glutaraldehyde Cross-linked Chitosan; Fe-CCTS: Fe- Cross-linked Chitosan; MCCTS: Magnetic Cross-linked Chitosan.

**Table 8:** Speciation of Cr(VI) and Cr(III) in environmental water samples (*n* = 3).

Water Samples	Cr(VI) (μg/L)				Cr(III) (μg/L)			
	Found	Spiked	Recovered	Recovery (%)	Found	Spiked	Recovered	Recovery (%)
Pond water	4.210	0.20	4.420	105	0.880	0.20	1.084	102
Lake water	2.250	0.20	2.458	104	0.420	0.20	0.618	99
Tap water	0.410	0.20	0.612	101	0.150	0.20	0.352	101

Data are expressed as mean of three replicates.

In order to apply the proposed method, speciation of Cr(VI) and Cr(III) in some environmental water samples (the pH value of water sample was adjusted to 4.0), including pond water, lake water, and tap water from Jiujiang University, China, were determined. At the same time, in order to validate the accuracy of the proposed method, different amounts of chromium were spiked in these environmental water samples. The results are given in Table 8. Good agreement was obtained between the added and the determined Cr(VI) and Cr(III) types. The recovery values calculated for the standard additions were in the range of 99–105%. The proposed method could thus be applied successfully for the separation and speciation of trace amounts of chromium in environmental water samples.

## 4. Conclusions

In this work, a new type of grafted chitosan adsorbent (HGCTS) was synthesized using2-hydroxyethyltrimethyl ammonium chloride. The adsorption of Cr(VI) on HGCTS was studied, and both adsorption equilibrium and adsorption kinetics were investigated. The adsorption isotherm could be well fitted by the Langmuir equation and the adsorption process could be best described by a pseudo second-order kinetic model. The result shows that the adsorption of Cr(VI) on HGCTS is favorable. Then, using HGCTS as adsorbent and FAAS as determination method, a novel and useful speciation technique for Cr(VI) and Cr(III) was offered. The presented procedure has been successfully applied for the separation and speciation of Cr(VI) and Cr(III) in environmental water samples with acceptable accuracy and precision.
